# Alterations in gut–kidney axis indicators and TMAO-related biomarkers in elderly patients with hypertensive nephropathy

**DOI:** 10.3389/fmed.2025.1671036

**Published:** 2025-09-04

**Authors:** Ziyi Li, Shi Yang, Xinyi Zhou, Yan Qing, Tiyan Zhang, Wenliu Xu, Wei Duan, Fajian Ren, Hua Deng, Wenjing Wang, Ming Li, Min Feng, Chaolong Rao

**Affiliations:** ^1^School of Public Health, Chengdu University of Traditional Chinese Medicine, Chengdu, China; ^2^Longquanyi Center for Disease Control and Prevention, Chengdu, China; ^3^Longquan Ping’an Community Health Service Center, Chengdu, China; ^4^Chengdu Association of Vision Protection and Health Promotion, Chengdu, China; ^5^Chengdu Center for Disease Control and Prevention, Chengdu, China

**Keywords:** gut microbiota, hypertensive renal dysfunction, trimethylamine N-oxide, 16S rRNA sequencing, cross-sectional study

## Abstract

**Introduction:**

Growing evidence suggests that gut microbiota may influence renal function via the gut–kidney axis. This study assessed gut microbial composition, metabolic indicators, and inflammatory markers in elderly individuals with varying degrees of hypertensive kidney involvement.

**Methods:**

Seventy participants were stratified into three groups: healthy controls, hypertensive without renal impairment, and hypertensive with chronic kidney disease.

**Results:**

The chronic kidney disease group exhibited elevated serum urea and creatinine and reduced eGFR, along with increased levels of KIM-1, NGAL, IL-18, TNF-α, IL-6, NF-κB, and FMO3. Urinary TMAO was significantly decreased in both hypertensive groups, while serum TMAO remained unchanged. Although α- and β-diversity indices were comparable across groups, compositional shifts were noted, including higher relative abundance of *Escherichia–Shigella* and *Haemophilus* and lower levels of *Faecalibacterium*. Correlation analyses revealed associations between specific genera and host metabolic or inflammatory markers, such as a positive correlation between *Enterobacter* and urinary TMAO, and inverse correlations between *Veillonella* and both eGFR and urinary TMAO. Functional prediction indicated increased amino acid metabolism in the chronic kidney disease group.

**Discussion:**

These findings suggest interrelated patterns involving gut microbial composition, toxin handling, and inflammatory status in elderly hypertensive individuals, supporting further investigation into microbiota-associated biomarkers within the framework of the gut–kidney axis.

## 1 Introduction

Hypertension ranks among the most common chronic non-communicable diseases globally and serves as a major contributor to cardiovascular complications and premature mortality (1). In China, population aging has driven a sustained increase in hypertension prevalence, particularly among older adults (2). Renal dysfunction frequently co-occurs with hypertension, and the two conditions are interlinked through a reciprocal mechanism: persistent hypertension accelerates renal decline, whereas impaired renal function compromises blood pressure regulation (3, 4). This bidirectional deterioration between hypertension and renal function is a critical factor in chronic kidney disease (CKD) progression and represents a principal pathway toward the development of end-stage renal disease (5).

Accumulating evidence suggests that alterations in gut microbial composition and metabolite profiles contribute to CKD development. Characteristic changes—such as reduced abundance of beneficial taxa like *Lactobacillus johnsonii*—have been linked to renal function and disease severity in microbiome-based studies (6). Bibliometric analyses have revealed a growing research focus on gut microbiota–CKD interactions, particularly involving microbial toxins, short-chain fatty acids (SCFAs), and uremic solute metabolism (7). Among these, tryptophan metabolism via kynurenine, serotonin, and indole pathways has emerged as a key link between microbial activity and host immune–epithelial homeostasis (8). In diabetic kidney disease, microbial-derived toxins such as p-cresyl sulfate (PCS) and indoxyl sulfate (IS), along with altered bile acid and SCFAs metabolism, contribute to glomerular injury and fibrogenesis (9). Recent studies further indicate that probiotic interventions may mitigate inflammation and preserve kidney function in dialysis patients (10), while microbiota-targeted strategies alleviate renal fibrosis during the transition from acute kidney injury to chronic kidney disease in aged models (11).

Beyond SCFAs, bile acid, and trimethylamine pathways, protein-bound and indole-derived solutes represent another major group of gut-derived toxins involved in CKD progression and immune–renal interaction. Protein-bound solutes such as PCS and IS—products of tyrosine and tryptophan fermentation, respectively—accumulate in patients with declining renal function and contribute to oxidative stress, inflammation, and tubulointerstitial fibrosis (12). Moreover, tryptophan-derived indole compounds, including indole-3-acetic acid (IAA) and indole-3-aldehyde (IAld), have been shown to activate the aryl hydrocarbon receptor (AhR) pathway, a key immunomodulatory axis implicated in glomerular injury and epithelial dysfunction (13). Recent studies have reported that reduced abundance of *Lactobacillus* and *Bifidobacterium* species is associated with lower serum levels of protective indole derivatives and increased activation of the AhR pathway in both membranous nephropathy models and patients with idiopathic nephropathy (14).

Recent findings highlight trimethylamine N-oxide (TMAO) as a critical gut-derived metabolite involved in CKD progression. Intestinal microbes convert dietary precursors such as choline and L-carnitine into trimethylamine (TMA), which is subsequently oxidized by hepatic flavin-containing monooxygenase 3 (FMO3) to produce TMAO (15). In CKD patients, reduced renal excretion and high consumption of animal-based foods contribute to elevated circulating TMAO levels, which have been associated with vascular dysfunction and increased cardiovascular risk (16, 17). Microbiota-targeted interventions, such as modulation of bacterial composition or dietary strategies, have shown promise in reducing TMAO levels and mitigating organ damage in preclinical settings (18). Experimental evidence further indicates that trimethylamine N-oxide (TMAO) exacerbates renal fibrosis and immune activation through vascular damage and inflammatory signaling cascades (19–21). Nuclear factor kappa B (NF-κB), a central transcriptional regulator of inflammation-related signaling cascades, is increasingly recognized as a key driver of immune activation and tissue injury in renal disease (22). Meanwhile, elevated levels of interleukin-6 (IL-6) and Tumor necrosis factor-alpha (TNF-*α*) have been associated with enhanced oxidative stress and leukocyte infiltration, thereby promoting nephron damage and fibrotic remodeling (23). Although certain population-based metabolomic studies have identified predictors of CKD risk beyond conventional measures such as estimated eGFR (24), most available evidence remains fragmented, as studies typically assess microbial metabolites, inflammatory mediators, or renal biomarkers in isolation. Moreover, existing studies have primarily focused on animal models or high-risk clinical cohorts, limiting their relevance to early renal impairment in community-dwelling elderly populations (25–31). Few investigations have integrated these mechanistic domains into a unified analytical framework appropriate for early-stage disease evaluation.

This fragmented approach limits a comprehensive understanding of gut–kidney axis disruption during the initial phase of disease progression. Among these fragmented indicators, TMAO has garnered particular attention. Notably, a recent meta-analysis reported that elevated TMAO levels have been linked to increased risks of all-cause and cardiovascular mortality among individuals with CKD (32), highlighting the need for integrated biomarker frameworks capable of capturing early pathological transitions.

This study aimed to characterize gut microbiota composition, TMAO-related metabolites, and selected serum biomarkers in elderly individuals with hypertension and early-stage hypertensive nephropathy. Comparative analysis across renal function subgroups was conducted to identify early biological alterations and potential associations within the gut–kidney axis in this high-risk population.

## 2 Materials and methods

### 2.1 Study populations

This cross-sectional study evaluated gut microbiota composition, TMAO levels, and metabolic parameters in three elderly groups: healthy elderly controls (EldGrp), hypertensive without renal impairment (HTNGrp), and hypertensive with chronic kidney disease (HTNCKDGrp). Participants were recruited from a local hospital and classified based on clinical records, recent physical examinations, and laboratory findings. A total of 70 elderly participants were enrolled and stratified into the three groups as follows: EldGrp (*n* = 24), HTNGrp (*n* = 23), and HTNCKDGrp (*n* = 23).

Hypertension in this study was defined based on a documented clinical diagnosis and the presence of antihypertensive treatment, as recorded in participants’ community health records. This approach was chosen to reflect the chronic hypertensive status of participants, rather than relying solely on transient blood pressure values. Participants were considered hypertensive if they had a previous clinical diagnosis of hypertension based on the 2018 Chinese Guidelines for the Management of Hypertension, which define it as systolic blood pressure ≥140 mmHg and/or diastolic blood pressure ≥90 mmHg measured on three separate, non-consecutive occasions in untreated individuals. Alternatively, participants receiving antihypertensive medications, as documented in medical records, were also classified as hypertensive regardless of their current blood pressure levels. Although blood pressure values were measured at the time of study enrollment, they were not used for group classification and are presented only as part of the clinical characteristics. Based on recent laboratory evaluations, CKD was defined as an eGFR ≤ 60 mL/min/1.73 m^2^ and a urinary albumin-to-creatinine (CREA) ratio between 30 and 300 mg/g. Individuals meeting the diagnostic criteria for hypertension and CKD were assigned to the HTNCKDGrp.

Eligible participants were aged 60 years or older and capable of completing study procedures and providing biological specimens. Primary exclusion criteria included a history of malignancy, recent cardiovascular events, major gastrointestinal disorders or surgeries, and recent use of antibiotics, probiotics, or other microbiota-modulating agents. Written informed consent was obtained from all participants. The study was conducted according to the guidelines of the Declaration of Helsinki and approved by the Ethics Committee of the Chengdu Center for Disease Control and Prevention (Approval No. 2024028).

### 2.2 Blood and urine sample collection and biomarker detection

Fasting venous blood (5 mL) and first-morning urine samples were collected at the community health facility and stored at −80°C under cold-chain conditions.

Routine serum biochemical parameters, including aspartate aminotransferase (AST), alanine aminotransferase (ALT), TBIL, UREA, CREA, eGFR, uric acid (UA), glucose (GLU), triglycerides (TG), total cholesterol (TC), high-density lipoprotein cholesterol (HDL-C), and low-density lipoprotein cholesterol (LDL-C), were analyzed using a fully automated biochemical analyzer (Model BS-450, Mindray Medical International Co., Ltd., Shenzhen, China; Registration No. 20152221143).

Serum and urine samples were submitted to Shanghai Enzyme-linked Biotechnology Co., Ltd. for enzyme-linked immunosorbent assay (ELISA)-based measurement of inflammation, renal injury, and toxin metabolism biomarkers. Analytes included TMAO, FMO3, IL-6, TNF-α, NF-κB, IL-18, KIM-1, NGAL, PERK, ANGII, and calcium/calmodulin-dependent protein kinase II (CaMKII). All ELISA tests were conducted using commercially available kits from mlbio.cn (Shanghai Enzyme-linked Biotechnology Co., Ltd.). According to the manufacturer’s general specifications, the kits have a typical detection limit of ~1.5 pg./mL, intra- and inter-assay coefficients of variation (CV) below 10%, and average recovery rates between 90 and 110% across serum and urine matrices. Concentration values were calculated from four-parameter logistic (4-PL) standard curves. Technical replicates were not included during testing.

### 2.3 Fecal sample collection and gut microbiota analysis

Fecal samples (1–3 g) were self-collected using sterile sampling kits and immediately transported on dry ice to the Chengdu Center for Disease Control and Prevention, where they were temporarily stored at −80°C. All samples were subsequently transferred in a single batch to Tsingke Biotechnology Co., Ltd. for gut microbiota profiling using 16S rRNA gene sequencing.

High-throughput paired-end sequencing was performed on the Illumina NovaSeq platform, targeting the V3–V4 hypervariable regions of the bacterial 16S rRNA gene. The primer sequences were as follows: Forward primer: 5′-ACTCCTACGGGAGGCAGCA-3′, Reverse primer: 5′-GGACTACHVGGGTWTCTAAT-3′.

Raw sequencing reads were permanently deleted by the sequencing provider after contract-mandated retention, and due to human subject confidentiality cannot be provided. Processed microbiome data (OTU tables, diversity indices, taxonomic annotation) are available upon reasonable request.

### 2.4 Statistical analysis

Statistical analyses of baseline demographic and serum biomarker data were performed using Statistical Package for the Social Sciences software (version 22.0). Continuous variables were screened for outliers using the robust regression and outlier removal (ROUT) method (*Q* = 1%) and excluded if identified. Normally distributed variables are expressed as mean ± standard deviation (SD) and compared across the three groups using one-way analysis of variance (ANOVA) followed by Tukey’s *post hoc* test. Non-normally distributed variables are presented as a median and interquartile range, with group comparisons conducted using the Kruskal–Wallis and Dunn’s multiple comparisons tests. Categorical variables are summarized as frequencies and percentages, with group differences assessed using the chi-square test or Fisher’s exact test, as appropriate. A two-sided *p*-value < 0.05 was considered statistically significant.

Spearman rank correlation analysis was performed to evaluate the associations between the relative abundances of selected microbial genera (e.g., *Escherichia–Shigella*, *Veillonella*) and host clinical parameters, including inflammatory cytokines (e.g., IL-6, IL-18), kidney function markers (e.g., serum creatinine, eGFR), and microbial metabolites (e.g., serum and urinary TMAO, FMO3). Correlation coefficients (r) and corresponding *p*-values were calculated based on genus-level relative abundance data and matched clinical measurements. Associations with a two-sided *p*-value < 0.05 were considered statistically significant. The results were visualized using a color-scaled heatmap representing the magnitude and direction of correlations.

Microbiota sequencing, diversity analysis, differential abundance assessment, and functional prediction were performed by a commercial provider (Beijing Tsingke Biotechnology Co., Ltd.). Raw sequencing data were first subjected to quality filtering using Trimmomatic (version 0.33) (33), followed by primer sequence identification and removal using Cutadapt (version 1.9.1) (34). Paired-end reads were then merged using USEARCH (version 10) (35), and chimeric sequences were removed using the UCHIME algorithm (version 8.1) (36). The resulting high-quality reads were used for downstream analysis.

Amplicon sequence variant (ASV) inference was performed using the Divisive Amplicon Denoising Algorithm 2 (DADA2) implemented in Quantitative Insights Into Microbial Ecology 2 (QIIME2) platform (version 2020.6) (37, 38). A relative abundance threshold of 0.005% of total sequences was applied to filter ASVs. Taxonomic classification was conducted based on the SILVA database (Release 138) (39). Annotation was performed using a hybrid approach: first, the classify-consensus-blast method was applied with ≥90% identity, ≥90% coverage, and ≥51% consensus threshold; for sequences not meeting these thresholds, the classify-sklearn method with a naive Bayes classifier and a confidence threshold of 0.7 was used as a supplementary tool.

Alpha diversity metrics were calculated using QIIME2 (version 2020.6), and beta diversity was analyzed to assess the compositional similarity between microbial communities across samples. Specifically, five alpha diversity indices were used: ACE and Chao1 indices estimate species richness within each sample; Shannon and Simpson indices reflect community diversity by incorporating both species richness and evenness; and PD_whole_tree evaluates phylogenetic diversity based on evolutionary relationships among taxa. Group comparisons of these indices were conducted using Student’s t-test, and visualization was performed using boxplots. Only statistically significant differences (*p* < 0.05) are indicated in the plots. Linear Discriminant Analysis Effect Size (LEfSe) was used to identify differentially abundant taxa between groups (40). This approach employs linear discriminant analysis (LDA) to estimate the contribution of each taxon to observed group differences, thereby identifying potential microbial biomarkers. Microbial co-occurrence network analysis was performed using Spearman rank correlation based on taxonomic abundance profiles. Only associations with |*r*| > 0.1 and *p* < 0.05 were retained for network construction. Phylogenetic Investigation of Communities by Reconstruction of Unobserved States 2 (PICRUSt2) was used for functional pathway prediction (41). Specifically, 16S rRNA feature sequences were aligned against reference sequences from the Integrated Microbial Genomes database to construct a phylogenetic tree and identify the closest reference organisms. Based on known gene content and abundance in these organisms, gene content in the sample was inferred. Functional pathways were then predicted using gene-to-pathway mapping from the Kyoto Encyclopedia of Genes and Genomes (KEGG) database. As a predictive tool, PICRUSt2 is limited by the coverage and accuracy of reference databases and does not reflect directly measured gene expression or activity.

## 3 Results

### 3.1 Group-wise characteristics and molecular indicators

**Figure 1:**
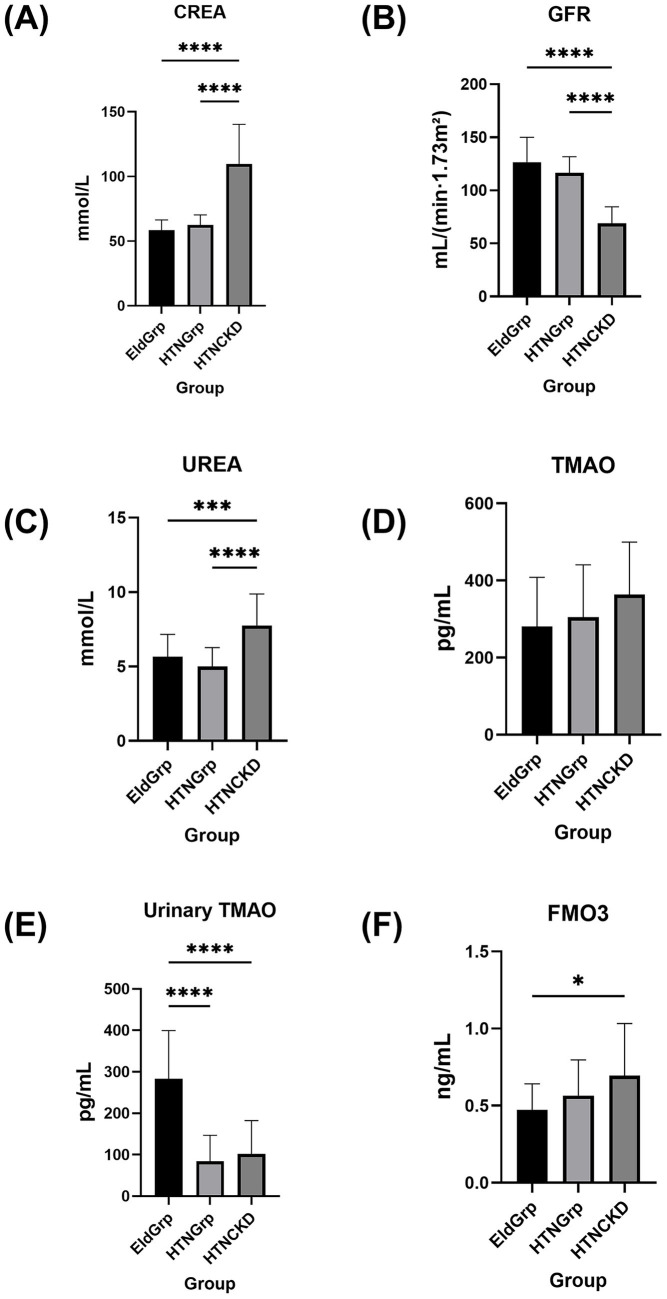
Group-wise comparisons of key renal and TMAO-related metabolic indicators among elderly participants. Group-wise comparisons of key renal and TMAO-related metabolic indicators among elderly participants. **(A)** CREA, **(B)** eGFR, **(C)** serum urea, **(D)** serum TMAO, **(E)** urinary TMAO, and **(F)** FMO3 levels were compared across three groups: healthy elderly controls (EldGrp), elderly with hypertension (HTNGrp), and elderly with hypertension and chronic kidney disease (HTNCKDGrp). Data are presented as mean ± standard deviation. Statistical comparisons were performed using one-way ANOVA followed by Tukey’s *post hoc* test. Significance levels:  *p < 0.05; ***p < 0.001; ****p < 0.0001.

Baseline demographic characteristics, biochemical parameters, and molecular biomarkers are summarized in Table 1. To highlight group-wise differences in key renal and metabolic markers, six representative indicators were visualized, including serum creatinine, CREA, eGFR, TMAO, urinary TMAO, and FMO3 (Figure 1). Among the demographic and lifestyle variables, only sex distribution differed significantly across groups (*p* = 0.0026); other factors such as age, body mass index (BMI), smoking, and alcohol use showed no significant differences. Several biochemical and molecular biomarkers, however, exhibited marked intergroup variation. The observed sex imbalance largely reflects the actual demographic profile of the community-based HTNCKD population. Several biochemical and molecular biomarkers, however, exhibited marked intergroup variation.

**Table 1:** Baseline characteristics and serum biochemical and biomarkers profiles in elderly participants.

Variable	EldGrp (*n* = 24)	HTNGrp (*n* = 23)	HTNCKDGrp (*n* = 23)	*P*-value
Age (years)	69.54 ± 5.61	74.61 ± 9.949	69.26 ± 11.67	0.1002
Sex (Male), *n* (%)	6 (25.0%)	6 (26.1%)	16 (69.6%)	0.0026**
BMI (kg/m^2^)	24.7 ± 3.554	25.03 ± 4.169	24.6 ± 3.117	0.917
Current smoker, *n* (%)	0 (0.0%)	2 (8.7%)	3 (13.0%)	0.1923
Current Alcohol Use, *n* (%)	3 (12.5%)	1 (4.3%)	6 (26.1%)	0.1095
Systolic Blood Pressure (mmHg)	149.2 ± 22.44	144 ± 23.08	142.7 ± 27.28	0.6266
Diastolic Blood Pressure (mmHg)	84.21 ± 12.48	81.61 ± 11.18	82.22 ± 14.32	0.7642
ALT (U/L)	17.73 ± 6.873ᵇ	18.02 ± 7.423ᵇ	24.70 ± 11.45ᵃ	0.0137*
AST (U/L)	21.78 ± 5.204	21.76 ± 5.921	23.80 ± 6.529	0.4375
TBIL (μmol/L)	12.11 ± 3.024ᵇ	11.95 ± 3.674ᵇ	15.97 ± 6.431ᵃ	0.0053**
UREA (mmol/L)	5.660 ± 1.493ᵇ	4.997 ± 1.272ᵇ	7.760 ± 2.113	<0.0001****
CREA (mmol/L)	58.89 ± 8.244ᵇ	60.77 ± 5.747ᵇ	142.8 ± 15.94ᵃ	<0.0001****
eGFR (min*1.73 m2)	126.0 ± 24.31ᵃ	120.1 ± 16.83ᵃ	51.36 ± 6.865ᵇ	<0.0001****
UA (mmol/L)	302.4 ± 50.01	325.2 ± 93.45	353.9 ± 112.3	0.1536
GLU (mmol/L)	5.722 ± 1.142	5.481 ± 0.9023	6.165 ± 1.403	0.1477
TG (mmol/L)	1.480 ± 0.7203	1.624 ± 0.7710	1.335 ± 0.5378	0.3743
TC (mmol/L)	4.787 ± 0.8606	4.866 ± 1.274	4.554 ± 0.9473	0.5743
HDL-C (mmol/L)	1.400 ± 0.3052	1.470 ± 0.3356	1.403 ± 0.2958	0.2931
LDL-C (mmol/L)	2.873 ± 0.7793	2.995 ± 0.9921	2.765 ± 0.8545	0.6764
TMAO (pg/mL)	280.5 ± 127.7	304.9 ± 136.0	363.5 ± 136.2	0.0992
Urinary TMAO (pg/mL)	283.1 ± 116.2ᵃ	84.39 ± 62.31ᵇ	101.6 ± 80.49ᵇ	<0.0001****
FMO3 (ng/mL)	0.4718 ± 0.1698ᵇ	0.5652 ± 0.2311ᵃᵇ	0.6943 ± 0.3391ᵃ	0.0180*
TNF-α (pg/mL)	4.165 ± 2.627ᵇ	5.169 ± 3.067ᵇ	10.40 ± 6.404ᵃ	<0.0001****
IL-6 (pg/mL)	1.272 ± 0.6794ᵇ	1.760 ± 0.4508ᵃᵇ	2.299 ± 1.141ᵃ	0.0004***
NF-κB (pg/mL)	37.43 ± 18.69ᵇ	41.15 ± 15.48ᵇ	59.59 ± 25.35ᵃ	0.0010***
KIM-1 (pg/mL)	44.54 ± 23.21^c^	59.48 ± 21.36ᵇ	77.88 ± 24.15ᵃ	<0.0001****
NGAL (pg/mL)	94.40 ± 52.83ᵇ	95.13 ± 37.75ᵇ	137.0 ± 43.24ᵃ	0.0025**
IL-18 (pg/mL)	49.46 ± 18.50ᵇ	47.21 ± 14.65ᵇ	73.67 ± 40.17ᵃ	0.0030**
PERK (pg/mL)	152.9 ± 79.00ᵇ	226.3 ± 105.8ᵃ	271.6 ± 100.3ᵃ	0.0003***
CaMKII (pg/mL)	336.9 ± 114.6	326.1 ± 115.5	371.5 ± 127.4	0.4062
ANGII (pg/mL)	64.34 ± 26.48ᵇ	79.55 ± 31.09ᵇ	119.8 ± 47.98ᵃ	<0.0001****

Data are presented as mean ± SD. Outliers were identified and excluded using the ROUT method (*Q* = 1%).

*p*-values were calculated using one-way ANOVA or Kruskal–Wallis tests. *Post hoc* comparisons were conducted using Tukey’s or Dunn’s tests, as appropriate.

*p < 0.05, **p < 0.01, ***p < 0.001, ****p < 0.0001.

Different lowercase letters (a, b, c) indicate statistically significant differences between groups (*p* < 0.05).

Liver enzyme levels demonstrated partial group-specific variations. ALT was significantly elevated in the HTNCKDGrp (*p* = 0.0137), whereas AST levels did not differ significantly (*p* = 0.4375). Total bilirubin (TBIL) remained significantly higher in the HTNCKDGrp (*p* = 0.0053). Renal function indicators followed a similar pattern: urea and CREA were significantly increased in the HTNCKDGrp (both *p* < 0.0001), whereas eGFR was significantly reduced (*p* < 0.0001), with the EldGrp exhibiting the highest values.

Regarding toxin metabolism, urinary TMAO levels were significantly decreased in both hypertensive groups relative to the EldGrp (*p* < 0.0001), whereas serum TMAO levels did not differ significantly. Conversely, serum FMO3 concentrations were significantly elevated in the HTNCKDGrp compared to the EldGrp (*p* < 0.05).

Inflammatory markers TNF-α, IL-6, and NF-κB were significantly elevated in the HTNCKDGrp relative to the other groups (*p* < 0.0001, *p* = 0.0004, and *p* = 0.0010, respectively).

Renal injury indicators, including KIM-1, NGAL, and IL-18 levels, were significantly increased in the HTNCKDGrp compared to the other groups (*p* < 0.0001, *p* = 0.0025, and *p* = 0.0030, respectively).

Regarding blood pressure-associated biomarkers, PERK and ANGII were significantly elevated in the HTNGrp and HTNCKDGrp compared to the EldGrp (*p* = 0.0003 and *p* < 0.0001, respectively). No significant group differences were identified for CaMKII.

### 3.2 Group-wise differences in gut microbiota composition

#### 3.2.1 Clustering patterns and interpretative analysis

**Figure 2:**
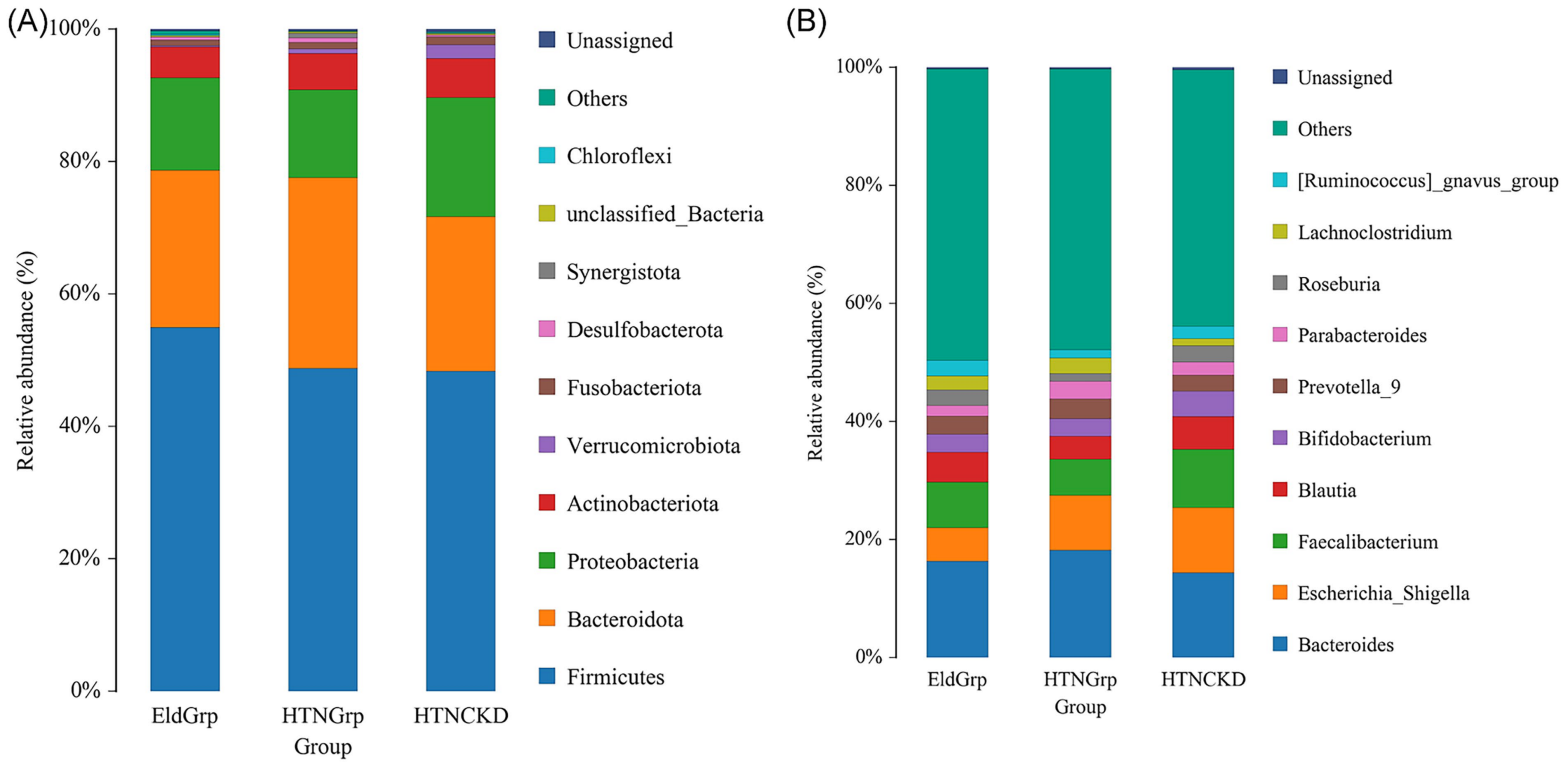
Relative abundance of gut microbiota at the phylum and genus levels across the three study groups. **(A)** Gut microbiota composition at the phylum level in EldGrp, HTNGrp, and HTNCKDGrp. The predominant phyla included *Firmicutes*, *Bacteroidota*, *Proteobacteria*, and *Actinobacteriota*. **(B)** Gut microbiota composition at the genus level, highlighting the relative abundance of dominant genera such as *Bacteroides*, *Faecalibacterium*, *Blautia*, and *Escherichia-Shigella*. Notable compositional shifts were observed in the HTNCKDGrp, characterized by increased *Escherichia-Shigella* and decreased beneficial genera such as *Faecalibacterium* and *Bifidobacterium*.

Figure 2A illustrates that the predominant phyla across all groups were *Firmicutes*, *Bacteroidota*, *Proteobacteria*, and *Actinobacteriota*. In the HTNCKDGrp, Firmicutes appeared less abundant, while *Proteobacteria* and *Actinobacteriota* showed relatively higher proportions compared to the EldGrp and HTNGrp.

While genus-level profiles (Figure 2B) varied visually across groups, statistical analyses did not indicate significant compositional differences. The HTNCKDGrp exhibited a higher relative abundance of *Escherichia-Shigella*, *Faecalibacterium*, *Bifidobacterium*, *Parabacteroides*, *Roseburia*, and *Lachnoclostridium* compared to the other groups. Conversely, *Prevotella_9* and *Bacteroides* were less abundant in the HTNCKDGrp relative to EldGrp and HTNGrp.

However, these apparent differences were not statistically significant based on Kruskal–Wallis tests (all *p* > 0.05). Detailed test statistics and groupwise medians are provided in Supplementary Table S1 (Sheet: Phylum_Level_Stats and Genus_Level_Stats).

#### 3.2.2 α-diversity and β-diversity across three groups

**Figure 3:**
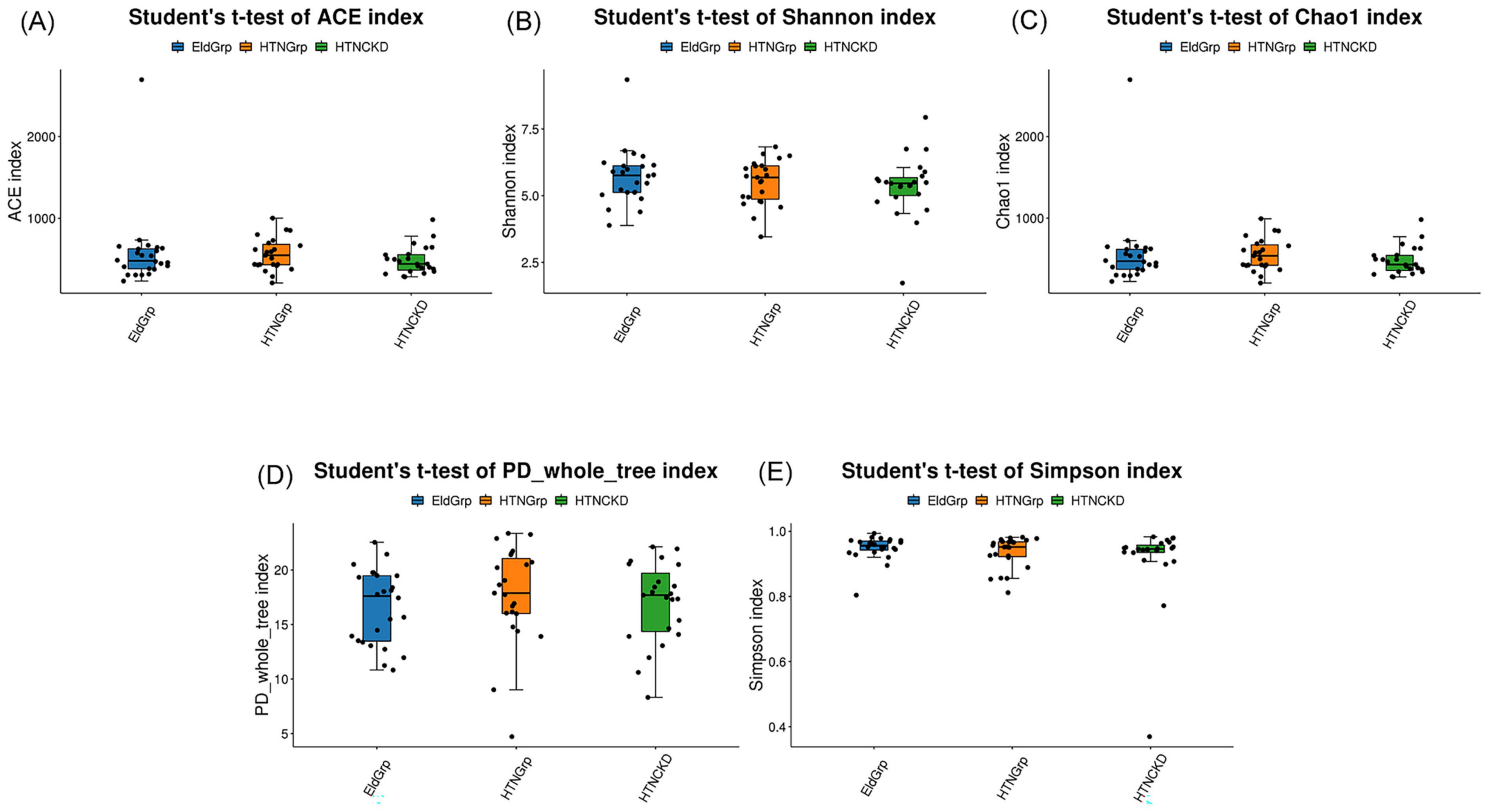
Alpha diversity indices of gut microbiota among the three study groups. Box plots compare gut microbial diversity and richness among EldGrp, HTNGrp, and HTNCKDGrp, based on the following indices: **(A)** ACE, **(B)** Shannon, **(C)** Chao1, **(D)** PD_whole_tree, and **(E)** Simpson. ACE and Chao1 indices estimate species richness; Shannon and Simpson indices reflect community diversity, incorporating both species count and distribution evenness; PD_whole_tree evaluates phylogenetic diversity based on evolutionary relationships. Group comparisons were performed using Student’s *t*-test. Only statistically significant differences (*p* < 0.05) are annotated in the figure; comparisons without annotations are not statistically significant. All calculations and plots were generated by the contracted bioinformatics service provider. The HTNCKDGrp exhibited a decreasing trend across all indices, though group-wise differences were not statistically significant.

Alpha diversity indices were employed to assess gut microbiota richness and diversity within each group. As shown in Figures 3A–E, no statistically significant differences were observed among groups. However, the HTNCKDGrp group showed a downward trend in both diversity-related indices (Shannon and Simpson) and richness-related indices (ACE, Chao1, and PD_whole_tree). Detailed test statistics, *p*-values, and group medians are provided in Supplementary Table S1 (Sheet: Alpha_diversity).

**Figure 4:**
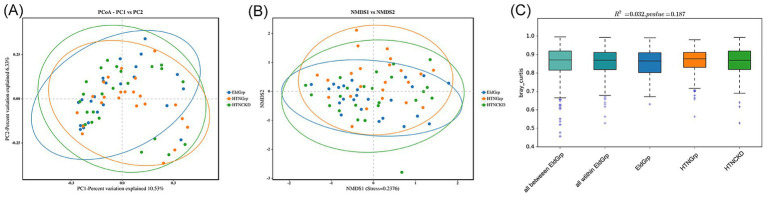
Beta analysis of gut microbiota among the three groups. **(A)** Principal coordinates analysis (PCoA) based on Bray–Curtis distance. Axes indicate the percentage of variation explained: PC1 = 10.53%, PC2 = 6.33%. **(B)** Non-metric multidimensional scaling (NMDS) plot based on Bray–Curtis distance. The stress value of the NMDS projection is 0.2376, indicating moderate ordination reliability. **(C)** Boxplots of Bray–Curtis distances representing intra- and inter-group comparisons. Ellipses in **(A,B)** represent 95% confidence intervals. PERMANOVA showed no statistically significant differences in microbial community composition among groups (*R*^2^ = 0.032, *p* = 0.187). “All within EldGrp” denotes distances among samples within the EldGrp group, and “All between EldGrp” denotes distances between EldGrp and the other two groups (HTNGrp and HTNCKDGrp).

Principal coordinate analysis (PCoA) based on Bray–Curtis distance was conducted to evaluate overall differences in gut microbiota structure among the three groups (Figure 4A). While the three groups showed substantial overlap, a slightly wider distribution was observed in the HTNCKDGrp, suggesting potential within-group variability (Figure 4A).

Non-metric multidimensional scaling (NMDS) based on the same distance metric revealed a similar clustering pattern (Figure 4B).

Permutational multivariate analysis of variance (PERMANOVA) demonstrated statistically non-significant differences among groups (*R*^2^ = 0.032, *p* = 0.187), indicating that the overall microbial community structure remained largely consistent across groups (Figure 4C).

#### 3.2.3 Differentially enriched taxa identified by LEfSe analysis

**Figure 5:**
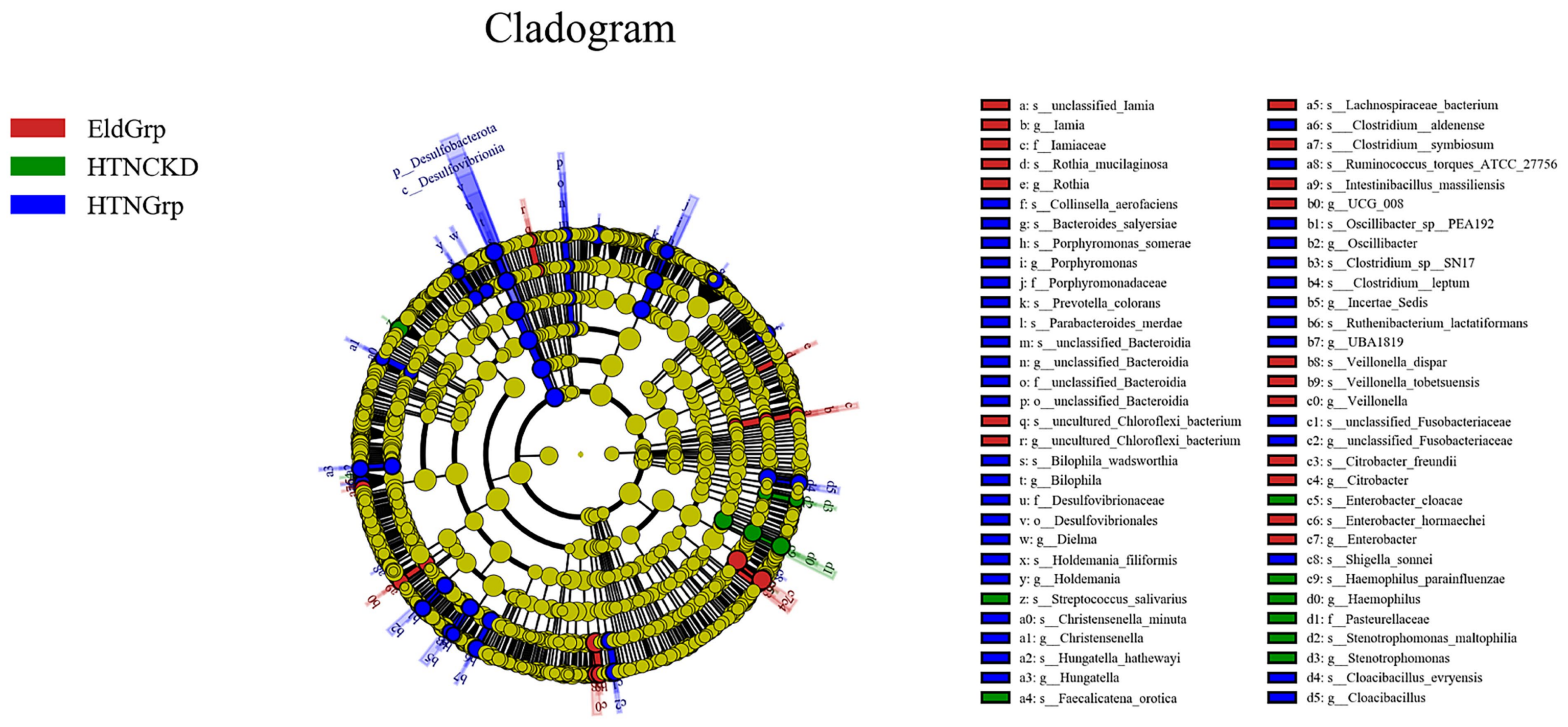
Differentially abundant taxa identified by LEfSe analysis. Cladogram illustrating the phylogenetic distribution of bacterial taxa differentially enriched among the three study groups. Nodes are colored by the group in which each taxon is overrepresented: red for EldGrp, green for HTNCKD, and blue for HTNGrp. The LDA threshold was set at 2.0.

**Figure 6:**
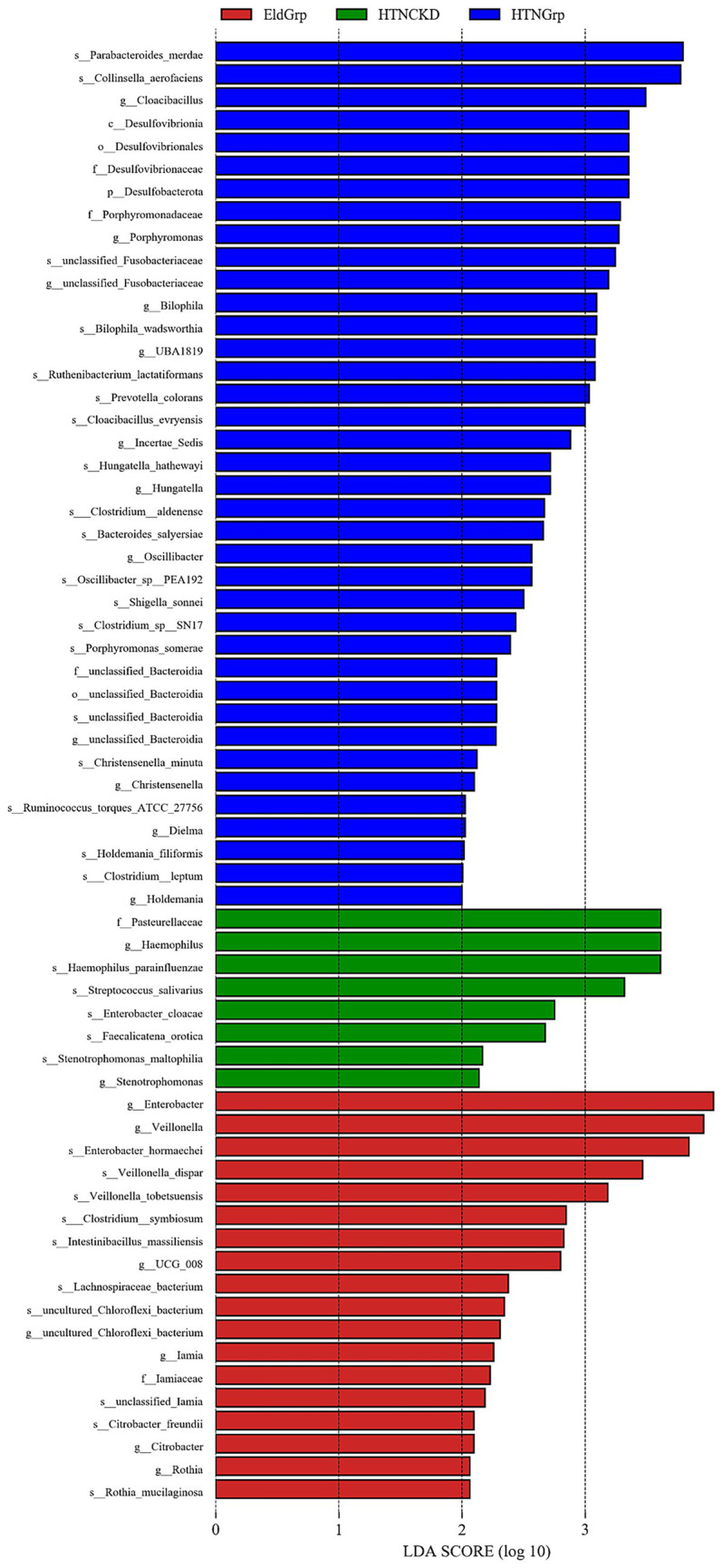
Histogram of LDA scores for differentially enriched genera among study groups. Histogram showing the LDA scores (log10) of bacterial taxa differentially enriched across the three study groups. Colors represent the enriched group: red for EldGrp, green for HTNCKD, and blue for HTNGrp. The LDA threshold was set at 2.0.

As shown in Figures 5, 6, LEfSe analysis identified several taxa with significant discriminative power among the three groups at the genus level. *Enterobacter* and *Veillonella* were identified as representative genera in the EldGrp, while *Bilophila* and *Christensenella* were enriched in the HTNGrp. In the HTNCKDGrp, *Haemophilus* was identified as a discriminative genus. Additional differentially abundant taxa at other taxonomic levels are presented in the corresponding figures.

#### 3.2.4 Correlation and network analysis

**Figure 7:**
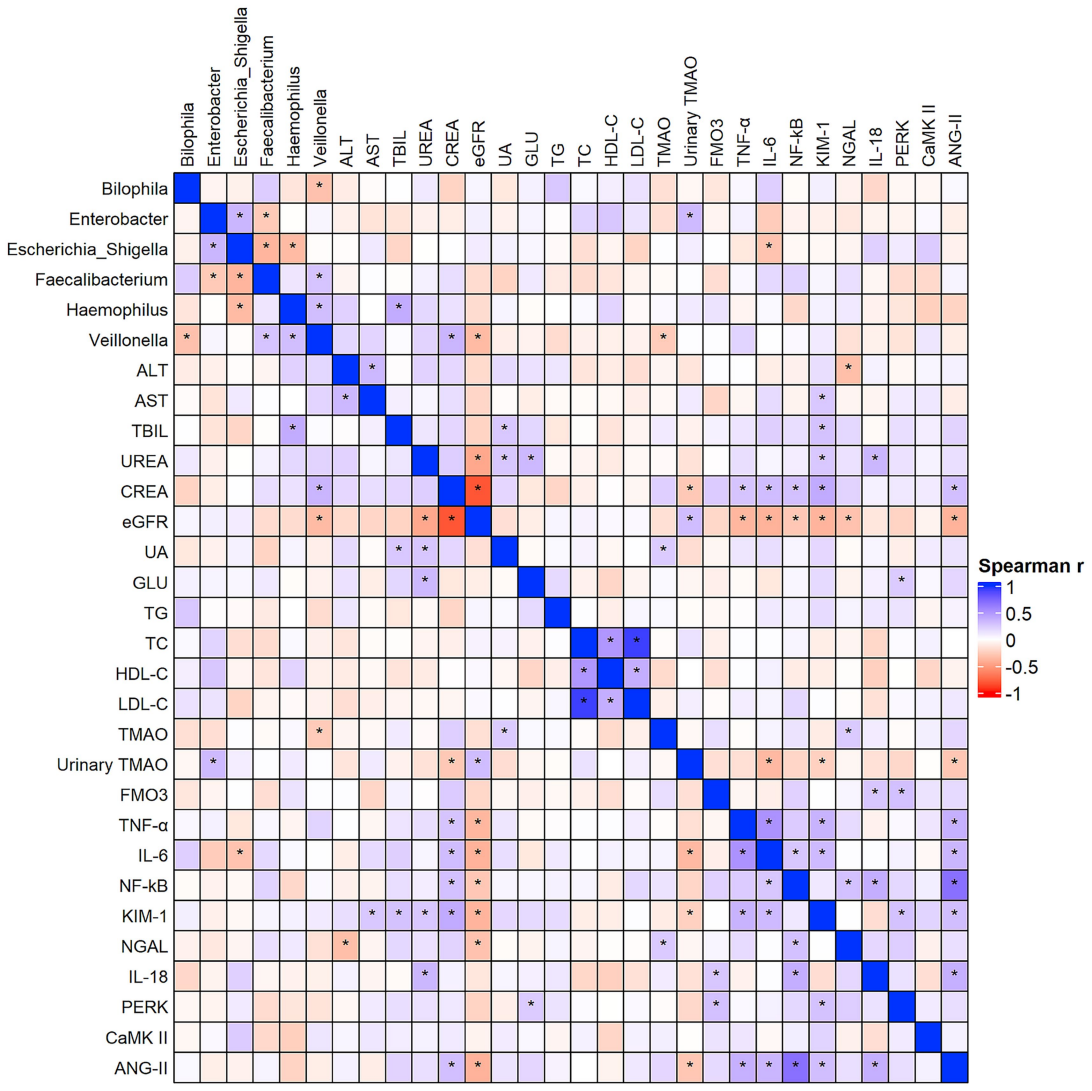
Spearman correlation analysis between key gut microbes, TMAO-related biomarkers, and clinical parameters. The color gradient represents the strength and direction of the correlation (blue for positive and red for negative). Full correlation coefficients are shown for exploratory purposes, while representative significant associations (*p* < 0.05) are detailed in the main text. “*”Indicates statistical significance at *p*< 0.05.

As shown in Figure 7, Spearman correlation analysis was performed for representative genera identified by LEfSe analysis. *Bilophila* was negatively correlated with *Veillonella* (*r* = −0.32, *p* = 0.03). *Haemophilus* showed a significant positive correlation with total bilirubin (TBIL) (*r* = 0.40, *p* = 0.003), a positive correlation with *Veillonella* (*r* = 0.31, *p* = 0.038), and was negatively correlated with *Escherichia–Shigella* (*r* = −0.35, *p* = 0.019). *Escherichia–Shigella* was negatively correlated with *Faecalibacterium* (*r* = −0.38, *p* = 0.01), positively correlated with *Enterobacter* (*r* = 0.35, *p* = 0.03), and negatively correlated with IL-6 (*r* = −0.31, *p* = 0.03). *Faecalibacterium* showed a positive correlation with *Veillonella* (*r* = 0.28, *p* = 0.05) and a negative correlation with *Enterobacter* (*r* = −0.28, *p* = 0.05). *Veillonella* was positively correlated with serum creatinine (CREA) (*r* = 0.35, *p* = 0.01) and negatively correlated with eGFR (*r* = −0.35, *p* = 0.01) and TMAO (*r* = −0.27, *p* = 0.046). *Enterobacter* was positively correlated with urinary TMAO (*r* = 0.33, *p* = 0.02).

Additionally, TMAO showed positive correlations with uric acid (UA) (*r* = 0.24, *p* = 0.04) and NGAL (*r* = 0.25, *p* = 0.04). Urinary TMAO was negatively correlated with CREA (*r* = −0.28, *p* = 0.02), IL-6 (*r* = −0.36, *p* = 0.02), KIM-1 (*r* = −0.24, *p* < 0.01), and ANG-II (*r* = −0.29, *p* = 0.02), and positively correlated with eGFR (*r* = 0.31, *p* = 0.01). FMO3 was positively correlated with IL-18 (*r* = 0.27, *p* = 0.04) and PERK (*r* = 0.30, *p* = 0.02).

Complete correlation coefficients and *p*-values are provided in Supplementary Table S1 (Sheet: Genus_and_Metabolite_Correlation).

**Figure 8:**
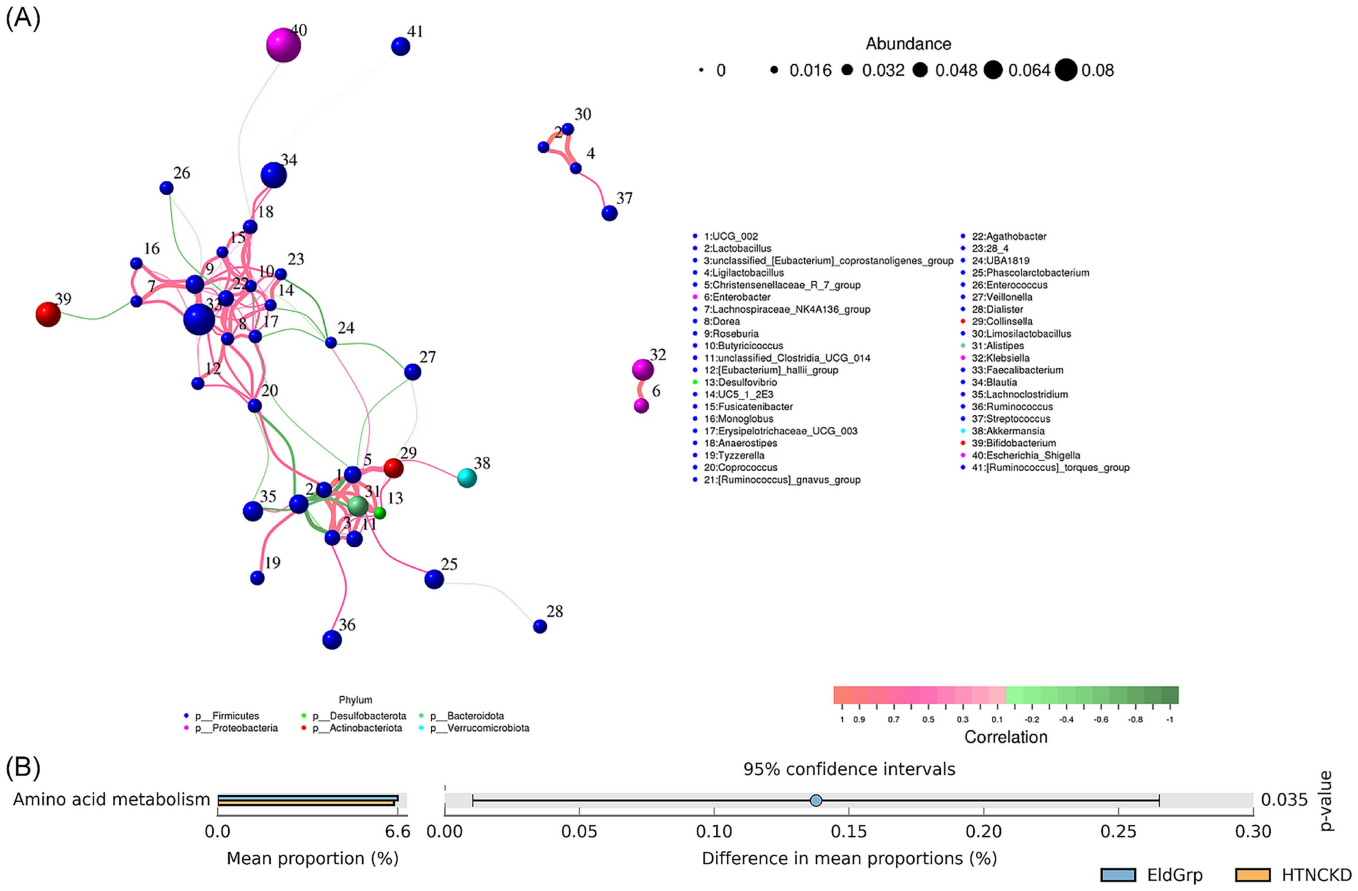
Correlation network and predicted functions of gut microbiota. **(A)** Co-occurrence network of gut microbial genera based on Spearman correlation analysis. Node size represents relative abundance, and edge color indicates positive (pink) or negative (green) correlations. **(B)** Predicted functional pathways of microbial communities across the three groups, based on PICRUSt2 analysis and presented at KEGG level 2. Among all pairwise comparisons, a significant difference was observed only between EldGrp and HTNCKDGrp.

A genus-level co-occurrence network was constructed to evaluate microbial interactions across all samples. Figure 8A presents correlations between the positive (pink edges) and negative (green edges) of multiple genera. Notably, *Bacteroides*, *Faecalibacterium*, and *Escherichia-Shigella* demonstrated high connectivity within the network.

At the KEGG Level 2 functional level, amino acid metabolism was significantly enriched in the HTNCKDGrp compared to the EldGrp (*p* = 0.035), while no other pathways showed significant differences (Figure 8B).

## 4 Discussion

This study compared EldGrp, HTNGrp, and HTNCKDGrp to capture the dynamic changes in gut microbiota and metabolites during the progression from hypertension to early renal impairment. This setup helps reveal how microbial and metabolic patterns shift as kidney disease develops.

This study observed differences in selected biochemical and clinical indicators among the EldGrp, HTNGrp, and HTNCKDGrp. In this study, elevated urea and serum creatinine levels, along with reduced eGFR in the HTNCKDGrp, were suggestive of renal impairment. These findings are consistent with previous reports indicating that hypertension contributes to CKD progression (42). No significant differences in renal function indicators, including eGFR and serum creatinine, were observed between the EldGrp and HTNGrp. This supports the classification of the HTNGrp as hypertensive individuals without overt renal impairment (43).

Regarding liver function, ALT and TBIL were significantly elevated in the HTNCKDGrp (*p* = 0.0137 and *p* = 0.0053, respectively), whereas AST levels remained unchanged (*p* = 0.4375). Although hepatic involvement is not a primary focus in hypertensive pathophysiology, previous studies have reported liver abnormalities in patients with hypertension and CKD, potentially mediated by systemic inflammation or dysregulated metabolic processes (44). These findings may reflect a broader systemic metabolic burden and suggest that hepatic markers could serve as supportive indicators of systemic stress in hypertensive nephropathy.

Multiple serum biomarkers associated with inflammation, cellular stress, renal injury, and toxin metabolism were significantly elevated in the HTNCKDGrp, reflecting activation of systemic stress and injury responses. Inflammatory cytokines TNF-*α*, IL-6, and NF-κB were markedly increased compared to the other groups. TNF-α has been closely associated with salt-sensitive hypertension and kidney injury, and is known to influence renal hemodynamics, excretory function, and the renin–angiotensin system (31). IL-6 has been identified as a downstream effector of angiotensin II, contributing to the expression of fibrotic mediators such as endothelin-1 and transforming growth factor β (TGF-β) (45), and along with TNF-α, is known to promote oxidative stress and immune cell infiltration in renal tissue (23, 27, 28, 46). NF-κB functions as a central transcriptional regulator that amplifies inflammatory cascades (22), and its excessive activation has been widely implicated in the progression of renal inflammation and fibrotic remodeling (47). Consistent with these observations, IL-18 was also elevated and has been linked to tubular injury and chronic inflammatory responses in the kidney (48).

Renal injury biomarkers KIM-1, NGAL, and IL-18 were elevated in the HTNCKDGrp, reinforcing evidence of nephron damage. KIM-1 is a sensitive indicator of early tubular injury, particularly in hypertensive renal damage, whereas NGAL, which is released in response to ischemic and inflammatory stimuli, reflects both acute and chronic nephron injury (27, 48–50). PERK, a well-recognized marker of endoplasmic reticulum stress (51), was found to be elevated in the HTNCKDGrp and may reflect intracellular stress responses secondary to hypertension-induced damage. CaMKII and ANGII were also significantly upregulated in the HTNCKDGrp. ANGII is the principal effector of the renin–angiotensin–aldosterone system (RAAS), promoting vasoconstriction, sodium retention, and pro-inflammatory signaling in renal tissues. CaMKII is activated downstream of ANGII and mediates calcium signaling, contributing to vascular remodeling and hypertension-induced renal damage (52–54). Collectively, these alterations suggest the activation of calcium signaling and the renin–angiotensin–aldosterone system, both of which are involved in vascular remodeling and renal impairment in hypertensive individuals (55). However, as this is a cross-sectional study, no causal relationship can be inferred from these associations, and the proposed mechanistic links remain speculative.

In addition to host biomarkers, we assessed host metabolites influenced by gut microbial activity. Although serum TMAO concentrations remained unchanged across groups, urinary TMAO levels were significantly reduced in both hypertensive groups compared to the EldGrp. Studies have shown that the renal clearance rate of TMAO is approximately twice that of CREA, indicating that its excretion is primarily dependent on glomerular filtration. Due to its small volume of distribution and lack of protein binding in plasma, serum TMAO levels may remain within the normal range during early-stage renal dysfunction, whereas urinary excretion may already be markedly reduced. This characteristic suggests that urinary TMAO could serve as a sensitive early indicator of renal function decline (56). The observed reduction likely reflects impaired renal clearance, as TMAO is primarily eliminated via glomerular filtration (57). Furthermore, reduced urinary TMAO in hypertensive individuals may indicate early alterations in renal handling, even in the absence of overt dysfunction. Simultaneously, hepatic FMO3 levels—the key enzyme catalyzing the conversion of microbial TMA to TMAO—were increased, suggesting enhanced host enzymatic capacity for TMA oxidation (58, 59). However, since TMA levels were not measured in this study, we cannot determine whether the observed FMO3 elevation reflects a compensatory response to increased microbial TMA production. Nevertheless, prior studies have demonstrated that gut microbiota composition—particularly the abundance of *Enterobacteriaceae*—can influence TMA synthesis, thereby potentially altering hepatic FMO3 activity (60). These findings support the potential value of urinary TMAO as an early and sensitive marker of renal impairment. Recent studies have highlighted that elevated circulating TMAO is associated with CKD progression and may serve not only as a biomarker but also as a potential therapeutic target (61).

Gut microbiota analysis identified compositional shifts in the HTNCKDGrp, with a relative decrease in *Firmicutes* and a trend toward increased proportions of *Proteobacteria* and *Actinobacteriota* at the phylum level. Similar alterations in phylum-level composition—characterized by reduced *Firmicutes* and increased *Proteobacteria* and *Actinobacteriota*—have been associated with impaired gut barrier function and systemic inflammation in previous studies (62–64). At the genus level, enrichment of *Escherichia-Shigella* and depletion of *Faecalibacterium* and *Bifidobacterium* were noted. *Escherichia-Shigella* has been associated with NOD-like receptor protein 3 (NLRP3) inflammasome activation and elevated TNF-*α* production, contributing to renal inflammation (65–67), whereas *Faecalibacterium* and *Bifidobacterium* exert SCFA-mediated anti-inflammatory effects (68). Decreased abundance of *Prevotella_9* and *Bacteroides* suggests impaired carbohydrate fermentation and SCFA production (69–71), while compensatory increases in *Parabacteroides*, *Roseburia*, and *Lachnoclostridium* may represent adaptive responses to preserve gut barrier integrity and immune homeostasis (72–76).

Although alpha and beta diversity metrics were not significantly different, the HTNCKDGrp exhibited a downward trend in Shannon, Simpson, and Chao1 indices, suggesting subtle reductions in microbial richness and ecological resilience. This pattern is consistent with previous findings that the gut microbiota in elderly individuals tends to maintain overall compositional stability unless exposed to strong perturbations, such as antibiotic use or severe disease. It has been reported that despite age-related declines in mucosal immunity, including reductions in taxon-specific immunoglobulin A, the gut microbiota composition remains largely stable in elderly individuals (77).

LEfSe analysis revealed distinct microbial signatures at the genus level across different clinical states in elderly participants. In the EldGrp, *Enterobacter* and *Veillonella* were identified as representative taxa, suggesting potential involvement in maintaining metabolic balance and microbial homeostasis. Although *Enterobacter* is typically low in abundance, it belongs to the Enterobacteriaceae family and has been implicated in anaerobic niche formation, vitamin synthesis, and inhibition of pathogens, potentially contributing to a stable microbial environment (78). *Veillonella*, a commensal anaerobe, utilizes lactate to produce short-chain fatty acids such as propionate and acetate, which are involved in immune regulation and energy metabolism (79).

*Bilophila* was enriched in the HTNGrp and is associated with bile acid dysregulation, high-fat diet-induced inflammation, and intestinal barrier impairment (80). Its expansion has also been linked to glucose metabolism disturbances and hepatic steatosis and may be partially modulated by probiotic interventions (81). In the HTNCKDGrp, *Haemophilus* was identified as a characteristic taxon. As a conditional pathogen, its abundance tends to increase under chronic inflammatory or immunocompromised conditions. A recent study using long-read sequencing showed a progressive rise in *Haemophilus* across different stages of CKD, suggesting its potential involvement in microbial imbalance and pro-inflammatory processes (82).

The observed microbial shifts may reflect a gradual reorganization of the gut community along the progression of hypertension and renal impairment, with a relative decrease in potentially beneficial taxa and an increase in pathobionts. Although mechanisms remain unclear, previous studies have noted that renal dysfunction often co-occurs with epithelial barrier disruption and microbial alterations, and experimental models suggest similar trends along the gut–kidney axis (83). Moreover, TMAO has been proposed as a metabolic link between cardiac and renal dysfunction, contributing to systemic inflammation and adverse outcomes in cardiorenal syndrome (84).

Correlation analysis revealed significant associations between representative genera and clinical parameters or microbial metabolites, suggesting their potential involvement in host metabolic regulation and inflammatory responses. *Veillonella* showed a positive correlation with serum creatinine and negative correlations with both eGFR and urinary TMAO, while *Enterobacter* was positively correlated with urinary TMAO, indicating their potential roles in toxin metabolism or kidney dysfunction. *Escherichia–Shigella* showed a negative correlation with IL-6, a positive correlation with *Enterobacter*, and a negative correlation with *Faecalibacterium*, suggesting a potential synergistic expansion under pro-inflammatory and dysbiotic conditions. This genus is commonly associated with microbial imbalance, inflammation, and epithelial barrier disruption, and its abundance has been shown to fluctuate dynamically across disease stages. Growing evidence indicates that gut microbiota contributes to oxidative stress and inflammation in CKD, exacerbating toxin translocation and fibrotic remodeling in the kidney (85). Notably, reduced levels of *Escherichia–Shigella* have been observed in end-stage renal disease patients undergoing hemodialysis, highlighting its possible role as a marker of gut dysbiosis (86). In addition, TMAO was positively correlated with uric acid and NGAL, while urinary TMAO showed negative correlations with creatinine, IL-6, KIM-1, and ANG II, and a positive correlation with eGFR. FMO3 was positively associated with IL-18 and markers of cellular stress. These findings support the potential role of microbially derived metabolites in linking gut dysbiosis to host inflammation and kidney injury. Experimental studies have demonstrated that TMAO can activate inflammatory pathways and promote the release of interleukins, contributing to renal inflammation and fibrosis (87).

Co-occurrence network analysis indicated that *Bacteroides*, *Faecalibacterium*, and *Escherichia–Shigella* had high centrality within the network, suggesting that these genera may occupy key ecological positions and that their interactions could be altered under disease conditions. A previous study in chronic kidney disease reported increased network complexity during disease progression, with *Oscillibacter* and *Veillonella* showing strong central roles, supporting the link between microbial network shifts and disease evolution (88). Functional prediction revealed a significant increase in amino acid metabolism pathways in the HTNCKD group, while other metabolic pathways remained relatively stable. This may reflect a limited capacity for functional resilience within the altered microbial community. Prior studies have shown that amino acid metabolism disorders in CKD are linked to the generation of protein-derived toxins and are accompanied by genomic structural changes (89). Multi-omics analyses further indicate that with advancing CKD severity, microbial composition and function undergo remodeling, including disruptions in glutathione and proline metabolism, suggesting that altered amino acid metabolism may play a central role in gut–host interactions (90).

Integrating these findings, our data suggest that the gut–kidney axis may be involved in the pathophysiological processes associated with hypertensive nephropathy. These changes observed in HTNCKDGrp may compromise gut barrier function. A previous study has shown that microbial products such as lipopolysaccharide can translocate across impaired gut barriers and activate innate immunity via TLR4–NF-κB signaling (91). This potential inflammatory cascade is supported by the elevated levels of circulating cytokines, including TNF-*α*, IL-6, and NF-κB (61), and may be further influenced by the accumulation of uremic toxins associated with reduced urinary TMAO and increased FMO3 levels (58, 59, 92). These alterations may be linked to tubular injury, interstitial inflammation, and cellular stress responses, as reflected by the elevated levels of KIM-1, NGAL, and PERK (48, 51, 52).

These findings raise the possibility that changes in gut microbial composition and host metabolic responses may be linked to systemic immune activation and toxin accumulation. Such interactions could be relevant to renal inflammation in hypertensive individuals. The gut–kidney axis may therefore represent a meaningful target for future research and therapeutic strategies in hypertensive nephropathy.

This study still has several limitations. First, the cross-sectional design precludes causal inference regarding the relationship between gut microbiota alterations and hypertensive nephropathy. Longitudinal or interventional studies are needed to clarify temporal relationships and potential mechanisms.

Second, although the overall sample size was relatively adequate, the number of participants in each group remained limited. This may reduce statistical power, particularly when detecting subtle changes in metabolic or inflammatory markers. Larger, multi-center studies are recommended to enhance the robustness of the findings.

Third, there were significant differences in sex distribution across groups. Because hypertensive nephropathy is more prevalent in males (93), eligible female participants were scarce and nearly all were included. Therefore, the imbalance largely reflects the actual demographic structure of the target population rather than selection bias. Nevertheless, sex-related differences in gut microbiota and inflammatory responses may still confound the results. Previous studies have shown that sex hormones and fat distribution can influence gut microbiota composition and its metabolic roles in obesity (94). Sex differences also modulate immune responses and microbial susceptibility to disease, suggesting that our findings may not fully capture sex-specific microbial interactions (95). Moreover, the current sample size was insufficient to perform sex-stratified analyses. In addition, the low healthcare utilization and disease recognition rates in community populations may have limited the inclusion of individuals with more advanced disease (96), potentially underestimating the magnitude of observed microbial differences in progressive hypertensive nephropathy. Although our current sample size precluded sex-specific comparisons, future studies should consider stratified recruitment to better examine potential gender-related differences in gut microbiota and immune responses.

Fourth, the classification of hypertension was based on diagnoses and antihypertensive medication use recorded in community health archives, rather than on-site blood pressure measurements. This approach aimed to reflect chronic hypertensive status more accurately. However, the study was conducted during a seasonal transition from summer to autumn, with notable temperature fluctuations. On the sampling day, some participants walked to the examination site early in the morning on an empty stomach and waited outdoors, possibly experiencing mild physical exertion, cold exposure, and psychological stress (97). These factors may have triggered transient elevations in blood pressure due to sympathetic activation. Given the age-related decline in vascular regulation, such short-term blood pressure increases are common in the elderly and may have obscured group differences in measured values. We also attempted a subgroup analysis based on antihypertensive medication use; however, the small number of cases precluded meaningful statistical comparisons. Additionally, information on blood pressure control status was not consistently available, preventing subgroup analysis based on hypertension control.

Fifth, although this study integrated gut microbiota, toxin metabolism, and inflammatory indicators, microbial functional inference relied primarily on PICRUSt2-based gene prediction. No validation was performed at the transcriptomic or metabolic flux level, and no *in vitro* or animal experiments were conducted. Future studies should incorporate metagenomic, transcriptomic, or metabolomic approaches, along with experimental models, to elucidate microbe-mediated pathogenic mechanisms.

Sixth, although key confounders were addressed in the study design, residual confounding from variables such as diet, medication adherence, and genetic background cannot be fully excluded. Future longitudinal studies should consider microbiota-targeted interventions—such as probiotics, prebiotics, and dietary modulation—to evaluate their potential in reducing inflammation, optimizing toxin metabolism, and mitigating renal injury. Fecal microbiota transplantation has shown promising therapeutic potential in preclinical models of CKD by modulating the renin–angiotensin system, restoring intestinal barrier integrity, and reducing inflammatory responses (98). Expanding participant diversity across ethnic and geographic backgrounds will also help improve the generalizability of the findings.

## 5 Conclusion

In elderly individuals with hypertensive nephropathy, elevated levels of NGAL, KIM-1, IL-18, TNF-α, IL-6, NF-κB, and FMO3 were observed, accompanied by reduced urinary TMAO concentrations and impaired renal function, as indicated by increased serum creatinine and decreased eGFR. These alterations were paralleled by shifts in gut microbial composition, including a higher relative abundance of *Escherichia–Shigella* and *Haemophilus*, and decreased levels of potentially beneficial taxa. Correlation and network analyses further revealed associations between representative genera and host inflammatory or renal markers, while functional prediction indicated enhanced microbial amino acid metabolism in the HTNCKD group. Although no causal inference can be drawn from this cross-sectional study, the findings highlight interconnected patterns involving inflammation, toxin metabolism, and gut microbiota along the early stages of hypertensive kidney injury. These results may provide a mechanistic basis for future research and support the potential utility of microbiota-related indicators—such as urinary TMAO and specific microbial taxa—as exploratory biomarkers in this population.

## Data Availability

The datasets presented in this study can be found in online repositories. The names of the repository/repositories and accession number(s) can be found: https://www.ncbi.nlm.nih.gov/bioproject/PRJNA1293923.

## Glossary

AhR: Aryl hydrocarbon Receptor
ALT: Alanine aminotransferase
ANGII: Angiotensin II
ANOVA: Analysis of variance
AST: Aspartate aminotransferase
ASV: Amplicon sequence variant
BMI: Body mass index
CaMKII: Calcium/calmodulin-dependent protein kinase II
CKD: Chronic kidney disease
CREA: Creatinine
DADA2: Divisive Amplicon Denoising Algorithm 2
eGFR: Estimated glomerular filtration rate
EldGrp: Healthy elderly control group
ELISA: Enzyme-linked immunosorbent assay
FMO3: Flavin-containing monooxygenase 3
GLU: Glucose
HDL-C: High-density lipoprotein cholesterol
HTNCKDGrp: Hypertensive with chronic kidney disease
HTNGrp: Hypertensive without renal impairment
IAA: Indole-3-acetic acid
IAld: Indole-3-aldehyde
IS: Indoxyl sulfate
IL-18: Interleukin-18
IL-6: Interleukin-6
KEGG: Kyoto Encyclopedia of Genes and Genomes
KIM-1: Kidney injury molecule-1
LDA: Linear discriminant analysis
LDL-C: Low-density lipoprotein cholesterol
LEfSe: Linear Discriminant Analysis Effect Size
NF-κB: Nuclear factor kappa B
NGAL: Neutrophil gelatinase-associated lipocalin
NLRP3: NOD-like receptor protein 3
NMDS: Non-metric multidimensional scaling
PCoA: Principal coordinate analysis
PCS: P-cresyl sulfate
PERK: Protein kinase R-like endoplasmic reticulum kinase
PERMANOVA: Permutational multivariate analysis of variance
PICRUSt2: Phylogenetic Investigation of Communities by Reconstruction of Unobserved States 2
QIIME2: Quantitative Insights Into Microbial Ecology 2
RAAS: Renin–Angiotensin–Aldosterone System
ROUT: Robust regression and outlier removal
rRNA: Ribosomal RNA
SCFAs: Short-chain fatty acids
SD: Standard deviation
TBIL: Total bilirubin
TC: Total cholesterol
TG: Triglycerides
TGF-*β*: Transforming Growth Factor Beta
TLR4: Toll-like receptor 4
TMA: Trimethylamine
TMAO: Trimethylamine N-oxide
TNF-*α*: Tumor necrosis factor-alpha
UA: Uric acid
